# Mechanistic decoding of triclosan-induced endometriosis via network toxicology, Mendelian randomization, and molecular docking

**DOI:** 10.1186/s40360-025-01030-x

**Published:** 2025-11-14

**Authors:** Bihong Xu, Maoqing Li, Xiaodong Zhao, Yu Qin, Ying Zhao

**Affiliations:** 1 Guangzhou University of Chinese Medicine, Guangzhou, China; 2 Wuyi Traditional Chinese Medicine Hospital of Jiangmen, Jiangmen, China; 3 Maoming Maternal and Child Health Hospital, Maoming, China; 4 The First Affiliated Hospital of Guangzhou University of Chinese Medicine, Guangzhou, 510000 China

**Keywords:** Triclosan, Endometriosis, Network toxicology, Mendelian randomization, Molecular docking, Environmental exposure

## Abstract

**Background:**

Triclosan, a widely used environmental contaminant with endocrine-disrupting properties, has been increasingly implicated in female reproductive disorders. However, the potential mechanistic link between triclosan exposure and endometriosis remains poorly understood.

**Methods:**

An integrative strategy combining network toxicology, Mendelian randomization (MR), and molecular docking was applied to explore the potential mechanistic association between triclosan and endometriosis. Toxicity and ADMET profiling of triclosan were performed using ProTox-3.0 and ADMETlab 2.0. Target prediction was conducted via STITCH and SwissTargetPrediction, while endometriosis-related genes were obtained from GeneCards, OMIM, and TTD. Common targets were subjected to PPI network construction and GO/KEGG enrichment analysis. MR analysis was performed to evaluate causality between IL1B expression and endometriosis risk. Molecular docking was used to validate the binding potential of triclosan to key proteins.

**Results:**

A total of 19 overlapping genes were identified between triclosan- and endometriosis-related targets. PPI network analysis revealed IL1B, SRC, EGFR, and KDR as hub genes. Enrichment analysis indicated significant involvement in MAPK signaling, VEGF signaling, and endocrine-related pathways. MR analysis supported a potential causal relationship between IL1B and endometriosis (MR-Egger OR = 2.66, *P* < 0.05). Docking simulations demonstrated stable binding of triclosan to all four hub targets, with binding energies ranging from − 5.3 to − 5.9 kcal/mol.

**Conclusion:**

This study provides mechanistic insights into the potential role of triclosan in endometriosis development, highlighting IL1B as a possible causal mediator. These findings contribute to a better understanding of environmental risk factors in endometriosis and offer potential molecular targets for future research.

**Supplementary Information:**

The online version contains supplementary material available at 10.1186/s40360-025-01030-x.

## Introduction

Endometriosis is a chronic, estrogen-dependent gynecological disorder characterized by the presence of endometrial-like tissue outside the uterine cavity [1, 2]. It affects approximately 10% of women of reproductive age and is often associated with pelvic pain, infertility, and a reduced quality of life [3]. Despite extensive research, the etiology and pathogenesis of endometriosis remain incompletely understood, involving a complex interplay of genetic, hormonal, immunological, and environmental factors.

Among the emerging environmental contributors, triclosan—a synthetic antimicrobial agent widely used in personal care products, plastics, and textiles—has raised increasing concern due to its endocrine-disrupting properties [4, 5]. Triclosan has been detected in urine, plasma, and even reproductive tissues, and its bioaccumulation may interfere with estrogen signaling, inflammation, and reproductive hormone regulation [6]. Epidemiological and experimental studies have suggested potential links between triclosan exposure and adverse reproductive outcomes, but its mechanistic involvement in endometriosis remains largely unexplored [7, 8].

In recent years, network toxicology has emerged as a powerful approach to systematically investigate chemical–disease associations through target prediction, protein interaction networks, and pathway enrichment analysis. Moreover, Mendelian randomization (MR), which leverages genetic variants as instrumental variables, enables causal inference from observational data, providing new insights into potential molecular mediators. Molecular docking further allows for validation of compound–target interactions at the structural level.

In this study, we employed an integrated strategy combining network toxicology, Mendelian randomization (MR), and molecular docking to decode the potential mechanisms by which triclosan may contribute to endometriosis. Unlike previous observational studies, our integrative framework enables a causal and structural interpretation of triclosan–endometriosis associations, providing novel mechanistic insights into its endocrine and inflammatory effects.

## Methods

The data in this study were obtained from various online database platforms, including PubChem, SwissTarget Prediction, CTD, GWAS, etc. The detailed databases used and the corresponding URLs are listed in Appendix A.

### Preliminary network analysis of triclosan toxicity

The toxicological and pharmacokinetic properties of triclosan were preliminarily evaluated using ProTox-3.0 and ADMETlab 2.0. ProTox-3.0 was used to predict oral toxicity, toxicity class, and potential organ-specific toxicities [9]. ADMETlab 2.0 provided absorption, distribution, metabolism, excretion, and toxicity (ADMET) profiles, including parameters such as lipophilicity, intestinal absorption, and hepatotoxicity [10]. These assessments served as the basis for subsequent network toxicology analysis.

### Gathering of triclosan targets

The molecular structure and SMILES representation of triclosan were first retrieved from the PubChem database [11]. Subsequently, two independent target prediction tools, STITCH and SwissTargetPrediction, were employed to identify potential human protein targets [12]. Both searches were restricted to Homo sapiens to enhance biological relevance. The results from both platforms were integrated, and redundant entries were eliminated to construct a consolidated set of triclosan-related genes for downstream analyses.

### Identification of endometriosis-related genes

Endometriosis-associated genes were collected from GeneCards, OMIM, and the Therapeutic Target Database (TTD) using “endometriosis” as the keyword. In the GeneCards database, genes with relevance scores above the median value were retained to ensure stronger disease relevance. The results from all three databases were merged, and duplicate entries were removed to generate a non-redundant gene set. This set was subsequently used to identify shared targets with triclosan for further network and enrichment analyses.

### Building a protein network and identifying key targets

Common targets shared by triclosan and endometriosis were imported into the STRING database to construct a protein–protein interaction (PPI) network, limited to Homo sapiens and filtered with a high-confidence interaction score threshold (>0.7) [13]. The resulting network was visualized using Cytoscape (v3.9.1), and topological parameters including degree, betweenness, closeness, and average shortest path length were calculated using the NetworkAnalyzer plugin. Genes ranking above the median in all metrics and exhibiting high degree centrality were defined as hub genes. In parallel, the MCODE plugin was used to identify densely connected clusters, further supporting the identification of key nodes. Based on these criteria, IL1B, SRC, EGFR, and KDR were selected as core targets for subsequent functional and mechanistic analyses.

### GO and KEGG enrichment analysis

Gene Ontology (GO) and Kyoto Encyclopedia of Genes and Genomes (KEGG) enrichment analyses were conducted using the clusterProfiler package in R. GO analysis encompassed three functional categories: biological processes (BP), molecular functions (MF), and cellular components (CC). KEGG pathway analysis was performed to identify signaling pathways potentially involved in the pathogenesis of triclosan-associated endometriosis. Terms with a *P*-value < 0.05 were considered statistically significant. The enrichment results were visualized using bar plots and bubble plots, highlighting the most relevant biological functions and pathways enriched among the overlapping target genes.

### Mendelian randomization analysis

MR is a genetic instrumental variable approach that utilizes single nucleotide polymorphisms (SNPs) derived from genome-wide association studies (GWAS) as proxies to estimate the causal effect of an exposure on an outcome.

To explore potential causal relationships between core targets and endometriosis, MR analysis was conducted using summary statistics from two independent GWAS datasets. For the exposure (IL1B expression), we used the eQTL dataset (OpenGWAS ID: eqtl-a-ENSG00000125538, Vosa U, 2018), which included 31,684 European individuals with 17,485 SNPs. For the outcome (endometriosis), we used the GWAS dataset (OpenGWAS ID: bbj-a-114, Ishigaki K, 2019), comprising 734 cases and 102,372 controls (total sample size = 103,106) of East Asian ancestry with 8,877,964 SNPs. Both datasets were aligned to the HG19/GRCh37 genome build. IL1B was designated as the exposure and endometriosis as the outcome, using GWAS summary statistics from datasets IDs eqtl-a-ENSG00000125538 and bbj-a-114, respectively, respectively. SNPs strongly associated with IL1B expression (*P* < 5 × 10⁸) were selected as instrumental variables (IVs). The inverse-variance weighted (IVW) method was employed as the primary analytical approach, with statistical significance defined as *P* < 0.05. To ensure robustness, additional MR analyses were conducted using MR-Egger, weighted median, simple mode, and weighted mode methods. All analyses were implemented using the TwoSampleMR package (v0.6.8) in R. These analyses were conducted to assess the potential causal effect of IL1B expression on the risk of endometriosis.

### Molecular docking for triclosan and core targets

Molecular docking was performed to assess the binding interactions between triclosan and the core target proteins identified from the PPI network. The 2D structure of triclosan was obtained in SDF format from the PubChem database. The three-dimensional structures of the target proteins (IL1B, SRC, EGFR, and KDR) were retrieved from the RCSB Protein Data Bank (PDB).

Docking simulations were conducted using CB-Dock2, an online docking tool that integrates cavity detection with blind docking based on the AutoDock Vina engine. Binding affinities were evaluated using Vina scores, with lower values indicating stronger predicted interactions. The docking results were used to validate the potential of the identified hub proteins as functionally relevant targets of triclosan.

## Results

### Preliminary network assessment of triclosan toxicity

**Fig. 1:**
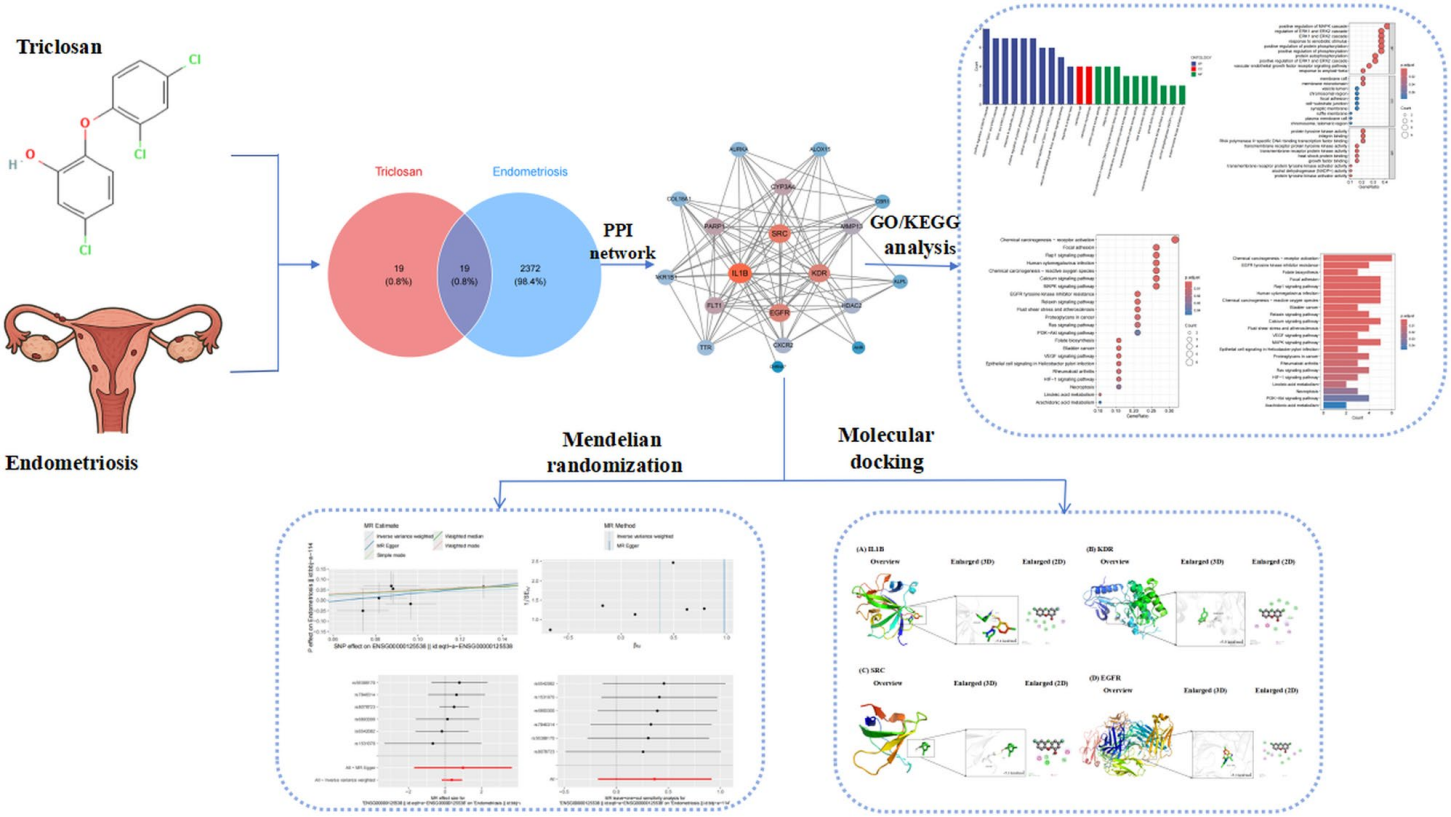
Workflow of the study. A stepwise pipeline integrating network toxicology, Mendelian randomization, and molecular docking was applied to explore the mechanistic association between triclosan exposure and endometriosis

The detailed flowchart of this study is presented in Fig. 1. Based on computational predictions, the toxicological profile of triclosan was systematically characterized. Toxicity models suggested low acute toxicity but identified potential risks including liver injury, poor bioavailability, and activation of endocrine-related pathways such as AhR and aromatase. These findings are consistent with its known biological activity and provide a basis for further mechanistic exploration. Full prediction results are available in the Table S1.

### Identification of targets of triclosan-induced endometriosis

**Fig. 2:**
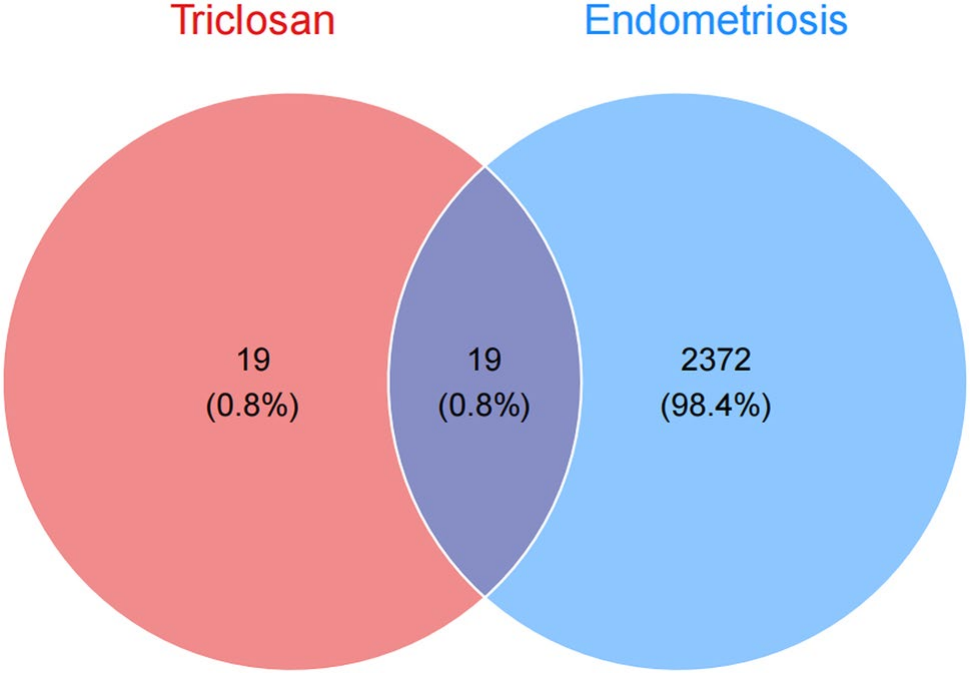
Venn diagram of the targets of Triclosan and Endometriosis

Potential targets of triclosan were predicted using STITCH and SwissTargetPrediction, yielding 38 unique genes after removing duplicates. Endometriosis-related genes were retrieved from GeneCards, OMIM, and the TTD, resulting in a total of 2,391 non-redundant genes. A total of 19 genes were found to overlap between the two datasets, representing potential mediators of triclosan-associated effects in endometriosis (Fig. 2). These targets were subjected to subsequent network and enrichment analyses. Details of the 19 triclosan-induced endometriosis targets are provided in the Table S2.

### PPI network construction and hub gene identification

**Fig. 3:**
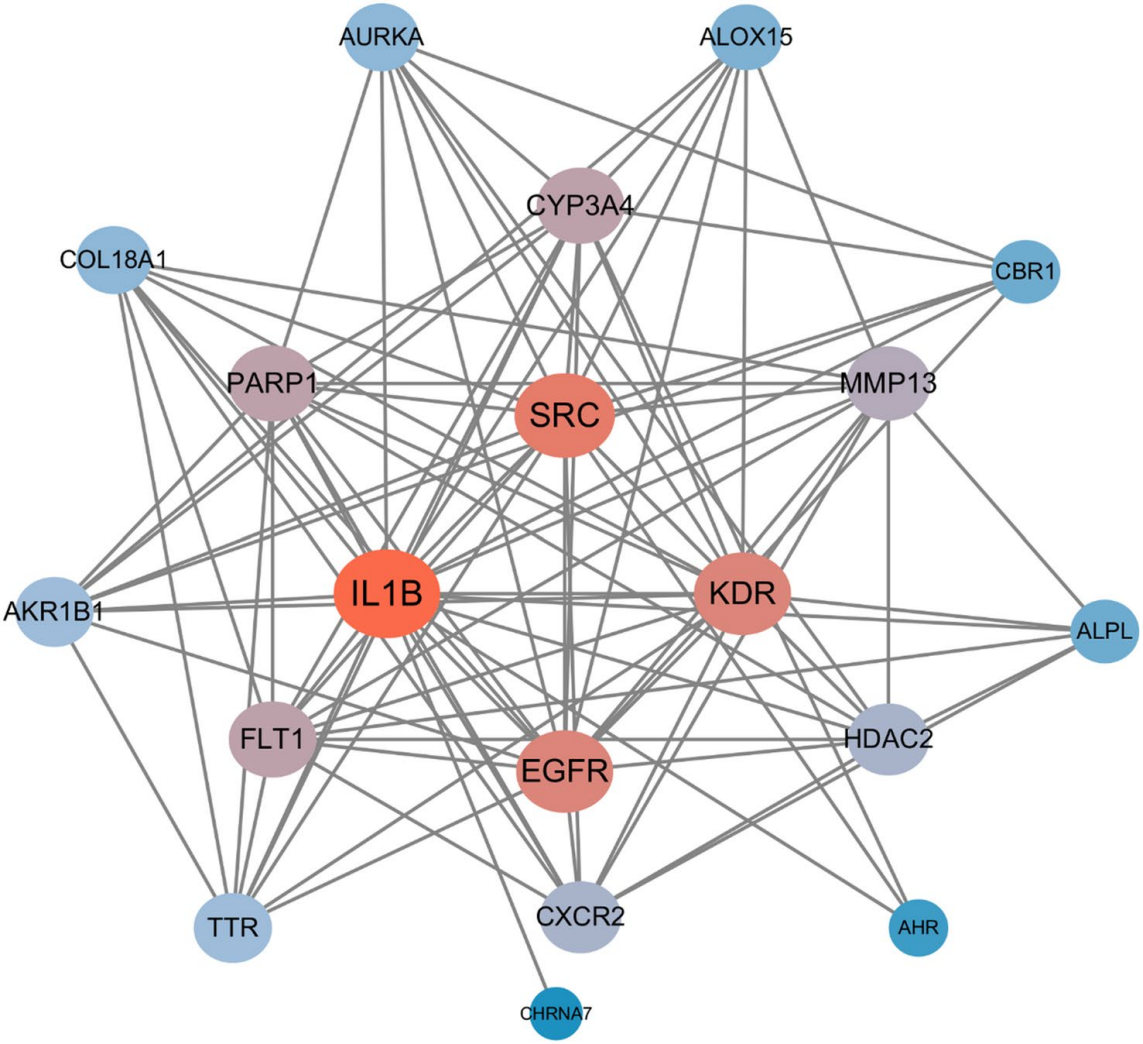
PPI network of intersecting targets with core genes highlighted

To explore the functional associations among the 19 shared targets of triclosan and endometriosis, a protein–protein interaction (PPI) network was constructed using the STRING database with a high-confidence threshold (> 0.7). The resulting network was visualized in Cytoscape, revealing dense interactions among most nodes (Fig. 3).

Topological analysis using the NetworkAnalyzer plugin was applied to calculate degree centrality and other network metrics. Four hub genes—IL1B, SRC, EGFR, and KDR—were identified based on high degree and topological prominence. These genes likely play central roles in triclosan-related modulation of endometriosis-related pathways and were selected for further Mendelian randomization and molecular docking analyses. A gene–gene interaction overview and co-expression heatmap are provided in the Supplementary Figures S1 and S2, respectively, to support the robustness of the network structure.

### GO and KEGG enrichment analysis of common targets

To explore the functional roles of the 19 overlapping targets between triclosan and endometriosis, GO and KEGG enrichment analyses were performed using the clusterProfiler package in R.

**Fig. 4:**
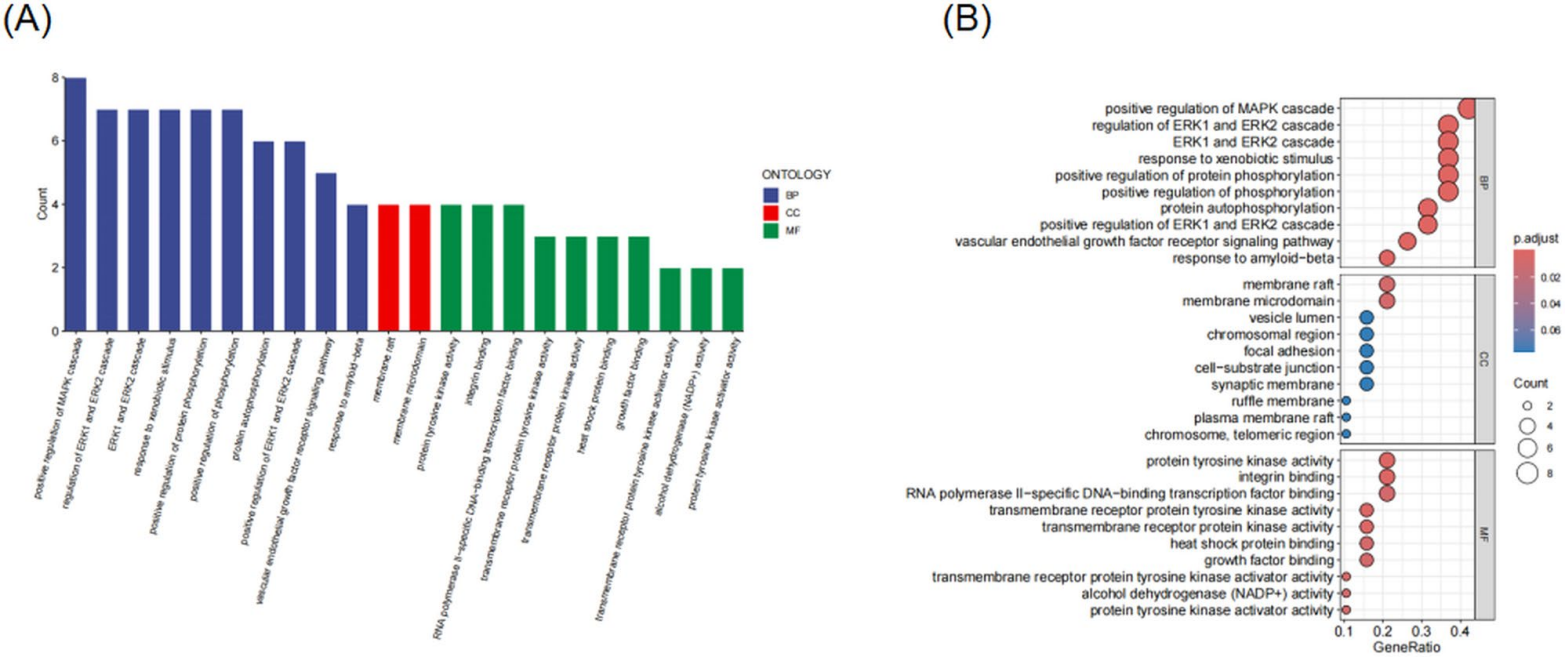
(**A**) Barplot of GO terms showing significantly enriched biological process (BP), cellular component (CC), and molecular function (MF). (**B**) Bubble plot displaying the top enriched GO terms, with size indicating gene count and color indicating adjusted *P*-value

GO analysis revealed that the enriched BP were primarily associated with the MAPK cascade, ERK1/ERK2 signaling, response to xenobiotic stimulus, and protein phosphorylation. CC terms such as membrane raft and microdomain were prominent, while MF categories were enriched for protein tyrosine kinase activity, transcription factor binding, and receptor binding (Fig. 4A, B).

**Fig. 5:**
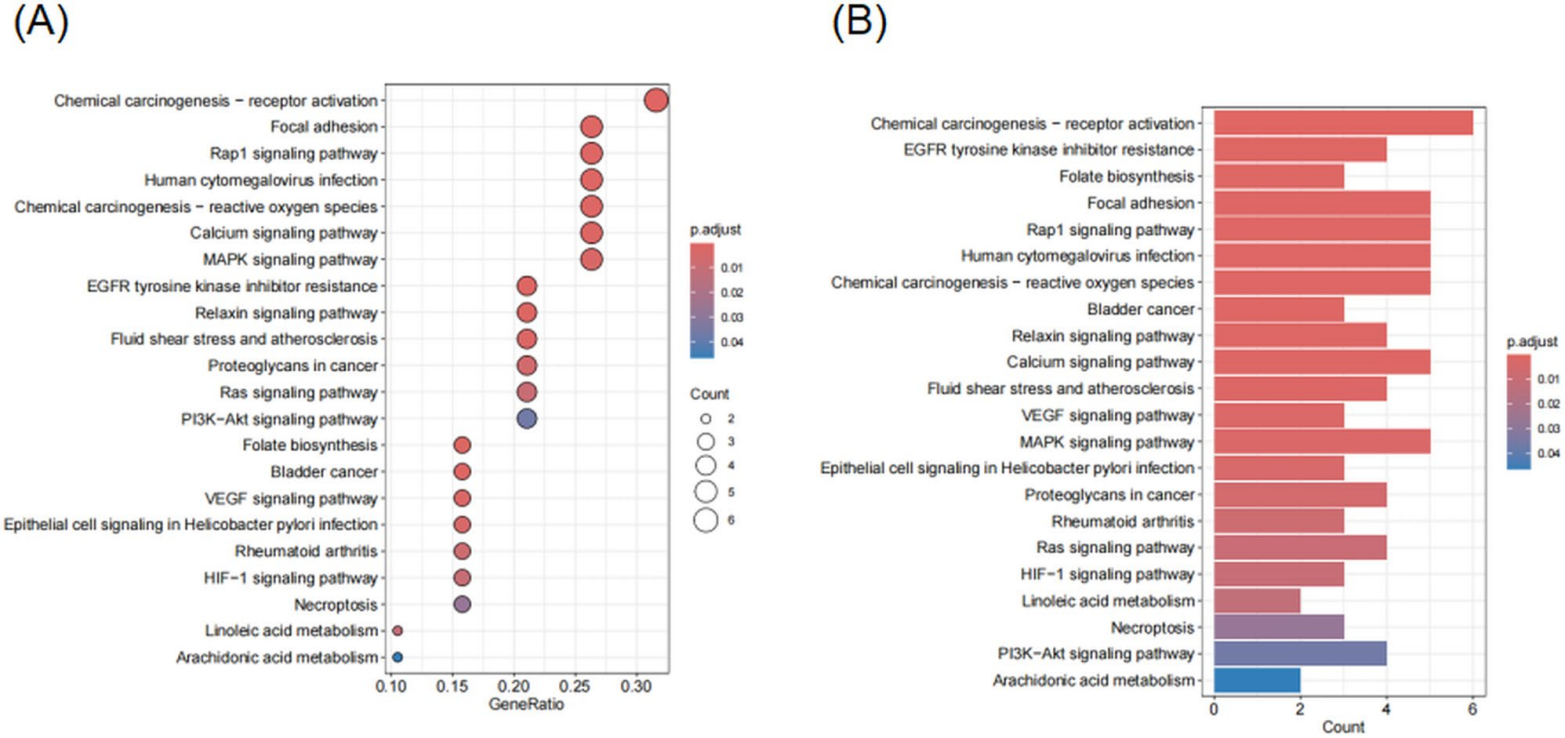
(**A**) Bubble plot of significantly enriched KEGG pathways related to metabolism, signaling, and disease. (**B**) Barplot of the top KEGG pathways, ranked by gene count and adjusted *P*-value

KEGG pathway analysis identified multiple signaling cascades involved in inflammation, cancer, and endocrine regulation. The most significantly enriched pathways included chemical carcinogenesis—receptor activation, focal adhesion, MAPK signaling pathway, VEGF signaling, PI3K-Akt signaling, and EGFR tyrosine kinase inhibitor resistance, among others (Fig. 5A, B).

These results indicate that the triclosan–endometriosis shared targets are strongly linked to inflammation-related and hormone-responsive pathways, supporting the hypothesis that triclosan may influence endometriosis progression through these mechanisms.

### Mendelian randomization analysis

**Fig. 6:**
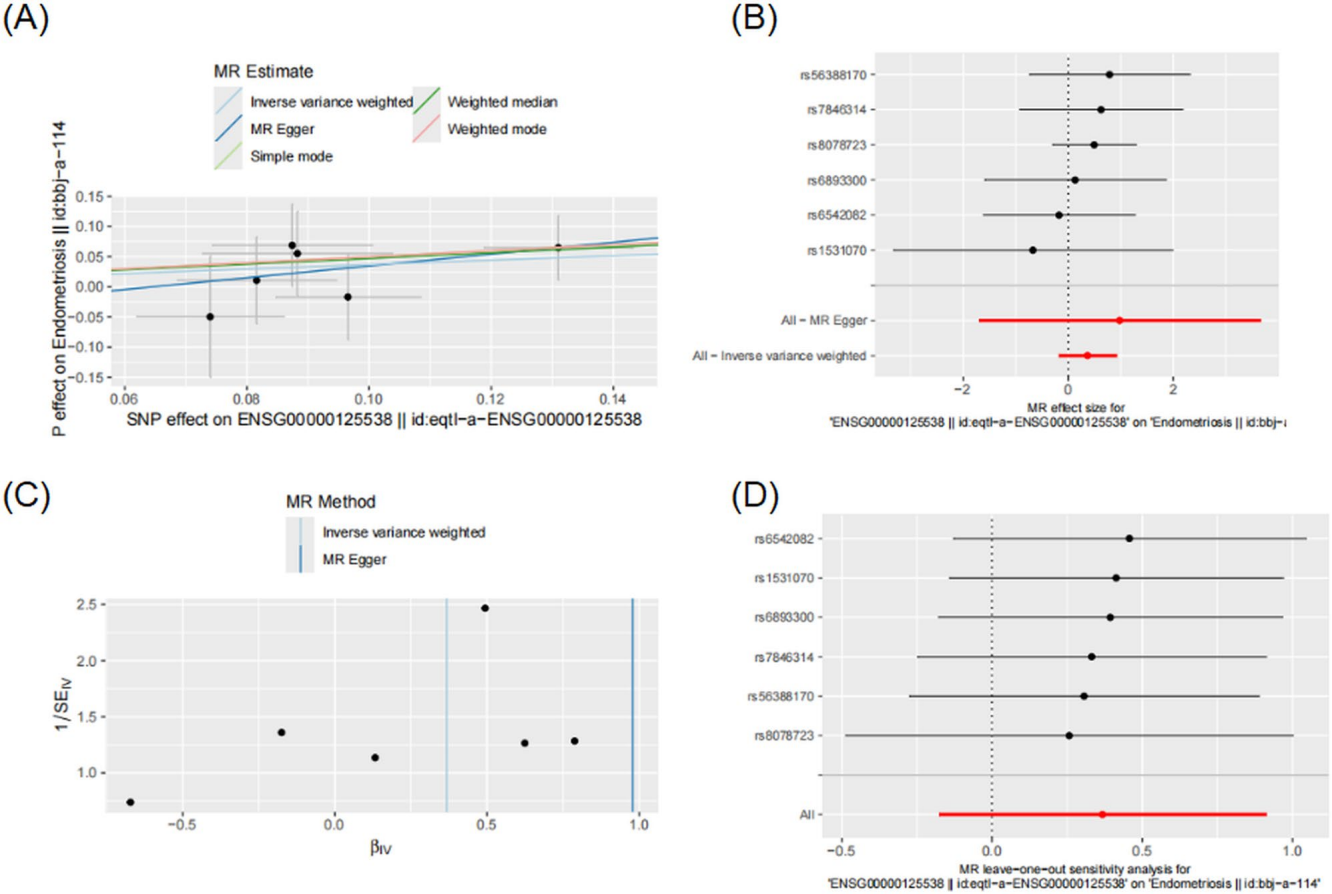
Mendelian randomization results assessing the causal effect of IL1B on Endometriosis. (**A**) Scatter plot of MR estimates using different methods. (**B**) Forest plot showing the effect of each SNP. (**C**) Funnel plot assessing heterogeneity. (**D**) Leave-one-out sensitivity analysis indicating no single SNP drives the overall effect

MR analysis was conducted to assess the causal relationship between IL1B expression and endometriosis. The exposure dataset (eqtl-a-ENSG00000125538) was based on 31,684 European individuals, while the outcome dataset (bbj-a-114) included 734 cases and 102,372 controls of East Asian ancestry. As shown in Fig. 6A, the scatter plot indicated a consistent positive association across all MR methods. The IVW method estimated an OR of 1.44 (95% CI: 0.84–2.49), while MR-Egger revealed a stronger effect (OR = 2.66, 95% CI: 1.32–3.51), both with *P* < 0.05 (Table 1).

Sensitivity analyses, including weighted median, simple mode, and weighted mode, yielded similar results. Forest plots (Fig. 6B) visualized the effect of each SNP, and funnel plot analysis (Fig. 6C) showed no evidence of significant heterogeneity or directional pleiotropy. The leave-one-out analysis (Fig. 6D) confirmed that the observed association was not driven by a single instrumental variable. These findings suggest a potential causal effect of IL1B on the risk of endometriosis.

**Table 1:** Mendelian analysis between IL1B and endometriosis

Mendelian Method	OR (95%CI)	*P*-value
Inverse-variance weighted	1.44 (0.84–2.49)	< 0.05
Weighted median	1.60 (0.84–3.06)	< 0.05
Simple mode	1.64 (0.60–4.53)	< 0.05
Weighted mode	1.64 (0.73–3.71)	< 0.05
MR Egger	2.66 (1.32–3.51)	< 0.05

### Molecular docking of triclosan with core targets

**Fig. 7:**
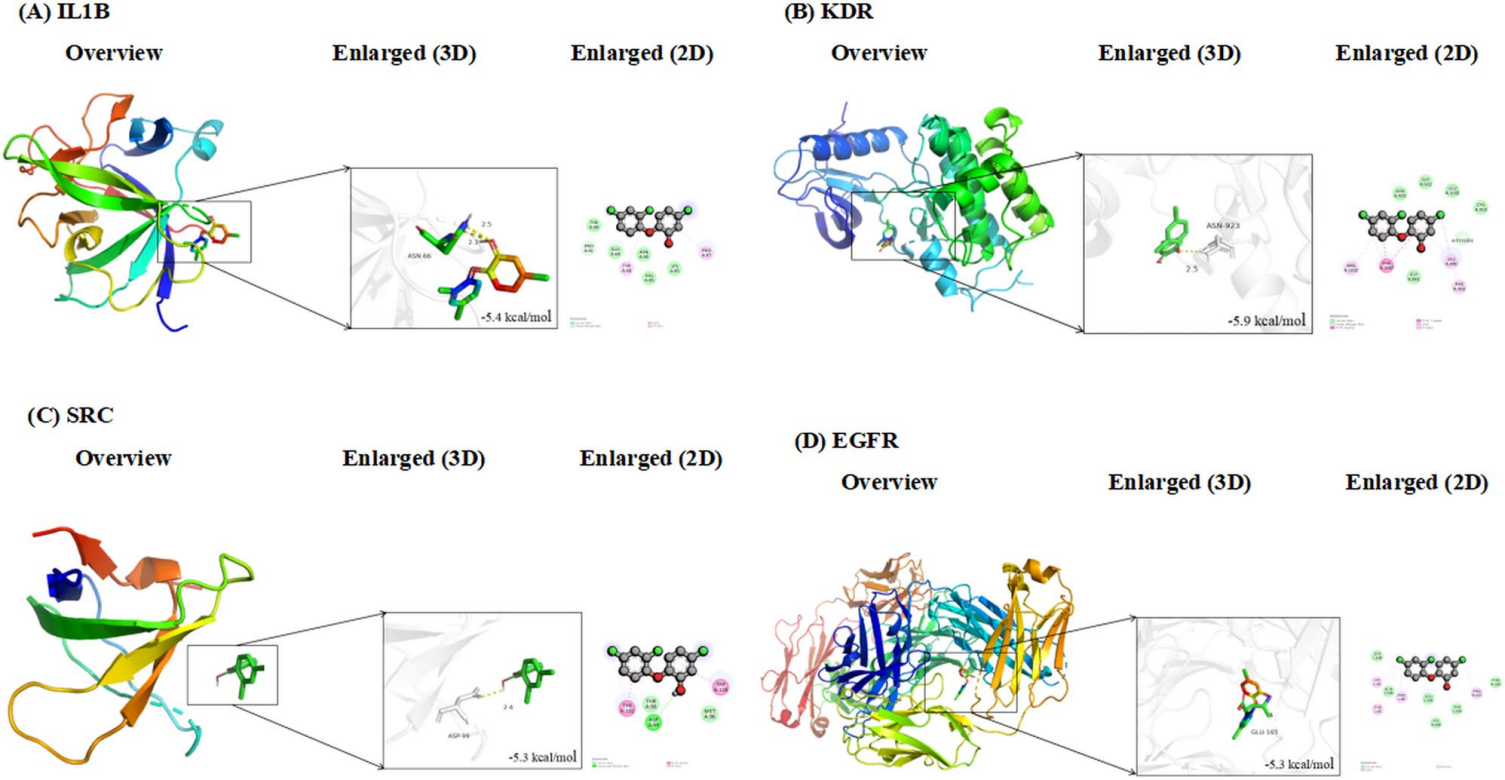
Molecular docking results in the lowest binding energy in each target protein with the DEHP. (**A**) Triclosan and IL1B, (**B**) Triclosan and KDR, (**C**) Triclosan and SRC, (**D**) Triclosan and EGFR

To validate the binding potential of triclosan to key proteins identified from the PPI network, molecular docking was performed with four hub targets: IL1B, KDR, SRC, and EGFR. The docking simulations revealed favorable binding affinities between triclosan and all four targets, with binding energies ranging from − 5.3 to − 5.9 kcal/mol (Fig. 7A–D).

Specifically, triclosan exhibited strong binding to KDR (–5.9 kcal/mol), followed by IL1B (–5.4 kcal/mol), EGFR (–5.3 kcal/mol), and SRC (–5.3 kcal/mol). Hydrogen bonding interactions were observed in the binding pockets, with key residues such as ASN923 (KDR), ARG66 (IL1B), ASP386 (EGFR), and ASP99 (SRC) contributing to ligand stability. Both 3D and 2D visualizations of the docking poses further confirmed these interactions. These results support the structural feasibility of triclosan binding directly to inflammation- and signaling-related proteins, reinforcing their potential roles in mediating triclosan-induced biological effects in endometriosis.

## Discussion

Compared with previous studies that primarily investigated the hormonal or toxicological impact of triclosan, our revised analysis integrates multi-omics data and genetic causal inference, offering a more comprehensive mechanistic interpretation of its potential role in endometriosis. This study integrated network toxicology, MR, and molecular docking to investigate the potential mechanistic link between triclosan exposure and endometriosis. Nineteen overlapping genes were identified, with IL1B, SRC, EGFR, and KDR emerging as hub targets. Enrichment analyses highlighted pathways including MAPK, PI3K-Akt, and VEGF, indicating that triclosan may influence inflammation and hormone-related processes relevant to endometriosis.

Previous studies have implicated triclosan in adverse reproductive outcomes, including reduced ovarian reserve and hormone disruption [14, 15]. Triclosan acts as an endocrine-disrupting chemical (EDC), capable of binding estrogen receptors and interfering with the hypothalamic–pituitary–gonadal axis [16, 17]. Pereira-Marostica and Ni demonstrated that triclosan induces hepatic enzymes and modulates xenobiotic metabolism, potentially disturbing systemic hormone levels [18, 19]. Our ADMET and ProTox predictions confirmed its capability to affect endocrine pathways and liver function, supporting these earlier findings.

Among the hub genes, IL1B stands out due to its well-documented role in endometriosis-related inflammation. Several studies have reported elevated IL1B expression in the peritoneal fluid, serum, and eutopic endometrial tissues of affected women [20]. IL1B promotes stromal proliferation and enhances COX-2 expression, facilitating prostaglandin-mediated lesion development [21]. Our Mendelian randomization analysis further supports a causal relationship between genetically predicted IL1B expression and endometriosis risk, aligning with prior GWAS-based observations [22].

The involvement of EGFR and SRC in endometriosis has also been noted. EGFR promotes endometrial cell proliferation and survival and is overexpressed in ectopic lesions [23]. EGFR inhibitors have shown therapeutic potential in experimental endometriosis models [24]. SRC functions as a central kinase linking growth factor and hormone signaling; Manek et al. identified its activation in early-stage endometriotic lesions, emphasizing its relevance in cellular migration and angiogenesis [25]. KDR (VEGFR2) is a key mediator of angiogenesis. Angiogenesis is essential for sustaining ectopic lesion growth, and KDR is upregulated in endometriotic stromal cells [26]. Our KEGG enrichment revealed significant overrepresentation of the VEGF signaling pathway, echoing the angiogenic profile of endometriosis [27].

Interestingly, the PI3K-Akt and MAPK pathways were also enriched. These cascades integrate inflammatory, hormonal, and stress responses, and are known to regulate survival of endometriotic cells [28]. Wang et al. showed that activation of MAPK/ERK promotes IL1B transcription, creating a positive feedback loop of inflammation [29].

Triclosan may influence these pathways directly. Molecular docking confirmed stable binding of triclosan to IL1B, EGFR, SRC, and KDR, with favorable energies and key interactions (e.g., ARG66 in IL1B, ASN923 in KDR). Previous docking and in vitro studies have reported similar results, suggesting that triclosan may mimic endogenous ligands and interfere with receptor function [30].

Together, these findings support a model where triclosan exposure activates IL1B and growth factor pathways, potentially exacerbating endometriosis through immune modulation, angiogenesis, and estrogen sensitivity. Our approach—integrating causal inference (MR), functional annotation, and structural validation—offers robust mechanistic insight beyond correlative observations.

The main strengths of this study include its multi-dimensional analytical framework, integrating toxicological prediction, Mendelian randomization, and molecular docking. This approach enhances causal inference and reduces potential confounding, thereby strengthening the evidence for IL1B’s involvement in endometriosis.

However, this study also has several limitations that should be acknowledged. First, the analyses rely on in silico predictions and publicly available datasets, which may lack tissue specificity or experimental validation. Second, although IL1B was prioritized as a causal gene, other overlapping targets may also contribute to triclosan’s effects and warrant further investigation. Finally, experimental or clinical validation is required to confirm the functional relevance of the identified molecular interactions.

## Conclusion

This study provides integrative evidence that triclosan may promote endometriosis through immune and growth-related pathways, with IL1B identified as a causal mediator. These targeted revisions have expanded the analytical scope and robustness of the findings, highlighting interleukin-1 beta (IL1B) as a potential causal mediator and confirming the reliability of triclosan–protein interactions through molecular docking validation. These findings offer new insight into the impact of environmental pollutants on gynecological health and highlight potential targets for therapeutic or preventative intervention.It should be noted that the exposure (IL1B expression) GWAS was conducted in a European population, whereas the outcome (endometriosis) GWAS was based on East Asian individuals. This trans-ancestry design may introduce population heterogeneity, and therefore the findings should be interpreted with caution. Furthermore, while MR provides evidence of potential causal relationships, experimental validation is required to confirm the underlying biological mechanisms.

## Supplementary Information

Below is the link to the electronic supplementary material.

Supplementary Material 1

## Data Availability

Data will be made available on request.

## Abbreviations

EGFR: Epidermal growth factor receptor
IL1B: Interleukin 1 beta
GO: Gene ontology
KEGG: Kyoto encyclopedia of genes and genomes
MAPK: Mitogen-activated protein kinase
MR: Mendelian randomization
PPI: Protein–protein interaction
SNP: Single nucleotide polymorphism
VEGF: Vascular endothelial growth factor
