# Supplementary material for: Mechanistic decoding of triclosan-induced endometriosis via network toxicology, Mendelian randomization, and molecular docking
Source: BMC Pharmacol Toxicol. 2025 Nov 14;26:191. doi: 10.1186/s40360-025-01030-x (PMC12619379; doi:10.1186/s40360-025-01030-x)
Supplement: Supplementary file 1 — Supplementary Material 1 [file 40360_2025_1030_MOESM1_ESM.docx]

Table S1. Website URLs for database and tools used in present study.

| Database | Website URL |
| --- | --- |
| PubChem | https://pubchem.ncbi.nlm.nih.gov/ |
| ProTox-II | https://tox.charite.de/ |
| ADMETlab | https://admetmesh.scbdd.com/ |
| STITCH | http://stitch.embl.de/ |
| Swisstargetprediction platform | http://www.swisstargetprediction.ch/ |
| OMIM | https://omim.org/ |
| GeneCards | http://www.genecards.org/ |
| Therapeutic Target Database | http://db.idrblab.net/ttd/ |
| STRING | https://string-db.org/ |
| PROTEIN DATA BANK | https://www.rcsb.org/ |
| Cytoscape | <https://cytoscape.org> |
| CB-DOCK | https://cadd.labshare.cn/cb-dock2/ |
| CTD | https://ctdbase.org/ |
| GWAS | https://gwas.mrcieu.ac.uk |

Table S2.

| Table S2. Potential targets of Triclosan-induced Endometriosis toxicity (19). | | | | |
| --- | --- | --- | --- | --- |
| IL1B | MMP13 | CYP3A4 | CBR1 | AHR |
| TTR | AKR1B1 | PARP1 | VEGFA | KDR |
| HDAC2 | AURKA | ALOX15 | CXCR2 | EGFR |
| FLT1 | CHRNA7 | ALPL | SRC |  |

| 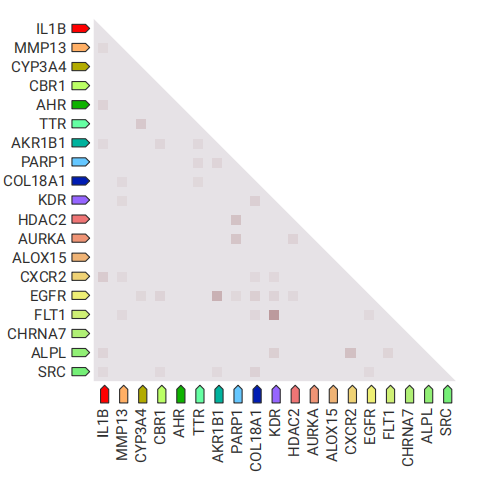 |
| --- |
| Figure S1. Co-expression relationships among the 19 shared targets visualized by STRING. |

| 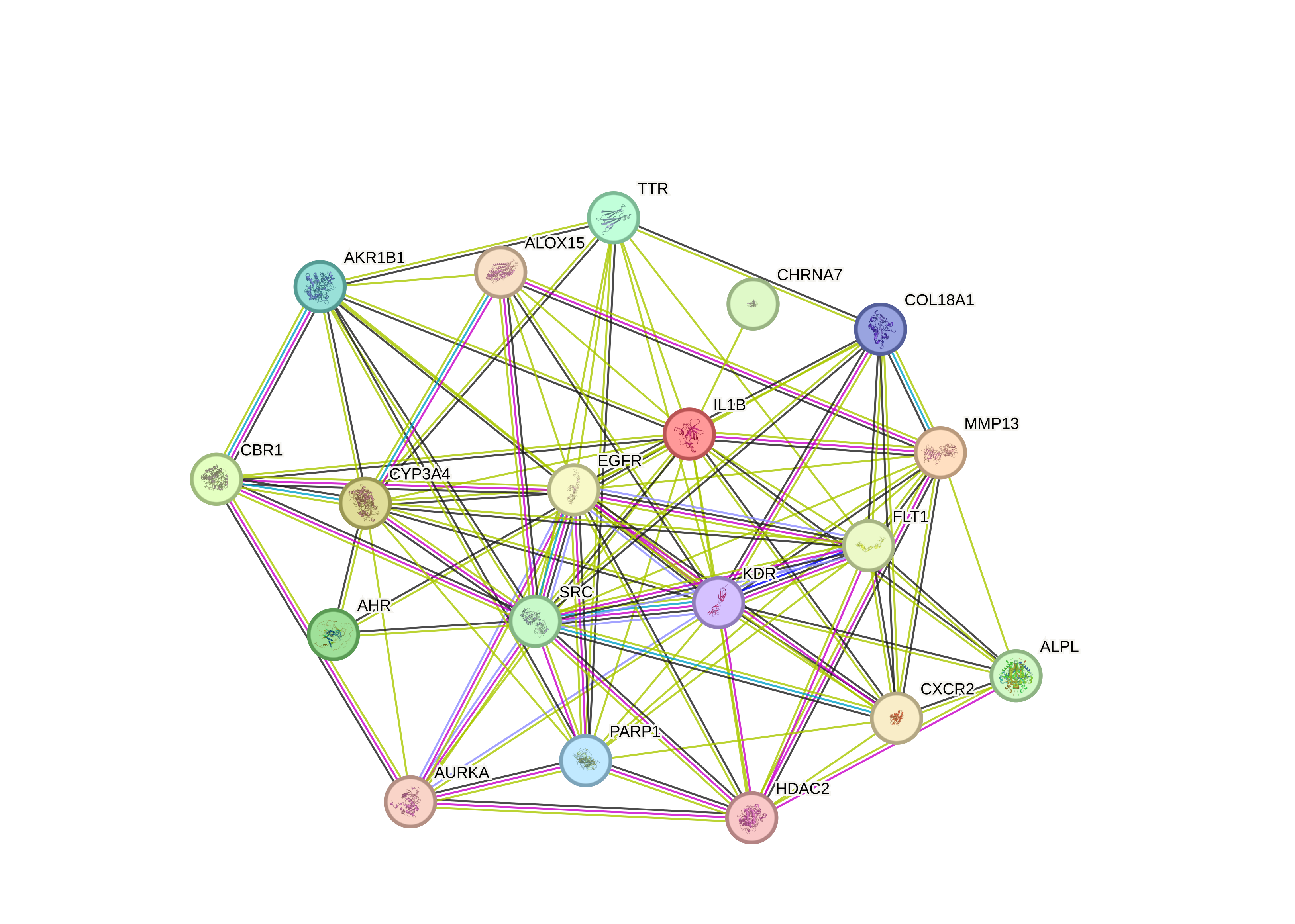 |
| --- |
| Figure S2. STRING network of functional associations among the 19 overlapping genes. |
